# Prediction of ^131^I uptake in lung metastases of differentiated thyroid cancer using deep learning

**DOI:** 10.3389/fendo.2025.1697233

**Published:** 2025-12-15

**Authors:** Hongjun Song, Manman Fei, Haoyi Tao, Zhongling Qiu, Chentian Shen, Xiaoyue Chen, Qiong Luo, Huajun She, Qian Wang, Lichi Zhang, Quanyong Luo

**Affiliations:** 1 Department of Nuclear Medicine, Shanghai Sixth People’s Hospital Affiliated to Shanghai Jiao Tong University School of Medicine, Shanghai, China; 2 School of Biomedical Engineering, Shanghai Jiao Tong University, Shanghai, China; 3 Department of Nuclear Medicine, Ruijin Hospital, Shanghai Jiao Tong University School of Medicine, Shanghai, China; 4 Department of Nuclear Medicine, Shanghai Tenth People’s Hospital, Tongji University School of Medicine, Shanghai, China; 5 School of Biomedical Engineering, ShanghaiTech University, Shanghai, China

**Keywords:** thyroid cancer, lung metastases, radioiodine therapy, deep learning, CNN - convolutional neural network

## Abstract

**Objective:**

An accurate assessment of ^131^I accumulation capacity in lung metastases of differentiated thyroid cancer (DTC) is pivotal for guiding radioiodine therapy and avoiding ineffective ^131^I administration. This study aimed to develop a deep convolutional neural network (DCNN) model to predict ^131^I uptake in lung metastases of DTC before radioiodine therapy.

**Methods:**

In this retrospective, multicenter, population-based cohort study, we collected chest CT image datasets for DTC patients with lung metastases from three hospitals in China. Pulmonary metastases were classified into two categories based on the post therapeutic ^131^I whole-body scan: ^131^I-avid (positive ^131^I uptake) and non-^131^I-avid (negative ^131^I uptake). For DCNN model development, patients were assigned to the primary dataset (140 patients with ^131^I-avid, 121 with non-^131^I-avid). For model validation, patients were assigned to the internal validation dataset (36 patients with ^131^I-avid, 23 with non-^131^I-avid), external validation dataset 1 (25 patients with ^131^I-avid, 18 with non-^131^I-avid), and external validation dataset 2 (23 patients with ^131^I-avid, 18 with non-^131^I-avid). Using these datasets, we assessed the performance of our model, ResNeSt50, and compared it with two models: Inception V3 and ResNet50.

**Results:**

Compared to Inception V3 and ResNet50, our model, ResNeSt50, demonstrated the highest prediction performance in the internal (area under the curve [AUC] = 0.722, 95% confidence interval [CI] = 0.716–0.725), external validation dataset 1 (AUC = 0.720, 95% CI = 0.691–0.749), and external validation dataset 2 (AUC = 0.731, 95% CI = 0.713–0.748).

**Conclusion:**

We developed a simple and robust DCNN model for predicting the ^131^I uptake in lung metastases of DTC before radioiodine therapy, which can provide improved screening for patients who may benefit from ^131^I therapy.

**Trial registration:**

Chinese Clinical Trial Registry (ChiCTR), ChiCTR1800018047. Registered on 28 August 2018.

## Introduction

Lungs are the most frequent site of distant metastases from thyroid cancer of follicular cell origin, with an incidence rate of 2% to 20% (1). The 10-year survival rate for patients with such metastases ranges from 25% to 85% (2–4). According to the post therapeutic radioactive iodine whole-body scan (^131^I-WBS), pulmonary metastases are classified as ^131^I-avid (positive ^131^I uptake) and non-^131^I-avid (negative ^131^I uptake). The term “^131^I-avid” refers to lesions that demonstrated higher radiopharmaceutical uptake than the physiological background of the mediastinum. The non-^131^I-avid category comprises lesions with no visible uptake on the post-theraputic whole-body scan (Rx-WBS) or, in cases of multiple pulmonary lesions, those with uptake evident in less than 10% of them. Radioiodine therapy remains the mainstay of treatment for ^131^I-avid lung metastases of differentiated thyroid cancer (DTC). However, in approximately 30% of cases, there is no obvious benefit, as DTC patients with pulmonary metastases may lose ^131^I accumulation capacity (radioactive iodine-refractory [RAIR]) (4). Generally, the 10-year survival rate of patients with RAIR-DTC is reported to be < 10%, and the prognosis is poor (5, 6), for which treatment with tyrosine kinase inhibitors (TKI) should be considered. Patients showing ^131^I accumulation should be treated with radioiodine therapy, whereas in the case of radioactive iodine-refractory patients, early identification of this subgroup is crucial to avoid unnecessary ^131^I treatment and, importantly, to prevent delays in other potentially effective therapies. However, the current lack of an effective, noninvasive method to predict ^131^I uptake in lung metastases before therapy can lead to potentially “blind” and ineffective treatments for some patients. Methods that may help predict ^131^I accumulation capacity in DTC lung metastases are urgently needed to facilitate individualized treatment. Computerized tomography (CT) scans accurately detect and diagnose lung metastases in DTC but are ineffective in identifying ^131^I-avid lung metastases. Artificial intelligence (AI), particularly deep learning algorithms, has achieved remarkable success and has already been successfully applied in the field of imaging diagnosis, classification, and prognosis owing to their advantages of being fast, accurate, and reproducible (7). AI models can discover high-order features and patterns in medical images that human experts cannot handle and can automatically perform quantitative assessments. Many models have even achieved performance comparable to human decision-making in recent applications (8, 9). Deep convolutional neural networks (DCNNs) are particularly recognized for their high performance in image recognition and their ability to accomplish complex visual recognition tasks (10). Deep learning has achieved state-of-the-art performance in automatic and accurate pulmonary nodule detection from CT scans (11). For example, DCNN can achieve an F-score of 85.5% for the classification of lung patterns on CT scans (12). Additional successes include lymph node metastasis prediction from primary breast cancer (13), diagnosis of thyroid cancer (14), and classification of renal cell carcinoma (15).

In this study, based on CT images, we aim to classify patients as ^131^I-avid and non-^131^I-avid using deep learning. Experiments validated the effectiveness of our classification model, with an area under the curve (AUC) of 0.722 (95% confidence interval [CI] = 0.716–0.725) in the internal validation dataset, 0.720 (95% CI = 0.691–0.749) in external validation dataset 1, and 0.731 (95% CI = 0.713–0.748) in external validation dataset 2.

## Materials and methods

### Study participants

This retrospective multicohort study was approved by the institutional review board (IRB) of Shanghai Sixth People’s Hospital Affiliated to Shanghai Jiao Tong University School of Medicine, Shanghai, China, and was undertaken according to the principles of the Declaration of Helsinki. Informed consent was waived by the IRB because of the retrospective nature of the study (Approval Number: 2018-KY-048 (K)). We accessed the medical record database from January 2017 to June 2022 to identify patients who had lung metastases, underwent total thyroidectomy for DTC, and received at least one ^131^I therapy.

### Diagnosis, classification, and treatment of DTC lung metastases

The diagnostic criteria for lung metastases of DTC were based on a previous study (4). A patient was considered to have pulmonary metastases if they met any of the following criteria: (i) confirmation of lung metastatic lesions through pathological examination of a biopsy specimen, (ii) presence of localized or diffused pulmonary ^131^I uptake on ^131^I-WBS in combination with pulmonary nodules on chest CT and elevated serum thyroglobulin (Tg) levels, or (iii) absence of pulmonary ^131^I uptake on ^131^I-WBS in combination with pulmonary nodules on chest CT and elevated serum Tg levels.

According to posttherapy (Rx-WBS) scanning after radioiodine therapy, lung metastases from thyroid cancer were classified as ^131^I-avid (positive ^131^I uptake) or non-^131^I-avid (negative ^131^I uptake). Lung ^131^I uptake was defined as “positive” when the lesion uptake was higher than the background physiological mediastinum uptake. Lesions without visible ^131^I lung uptake on Rx-WBS or with uptake in < 10% of multiple pulmonary lesions were defined as the “negative” group (16, 17). Regarding interrater discordance, two physicians initially interpreted the scans independently. In cases of disagreement, they reevaluated the scans together to reach a consensus.

Patients were instructed to follow a low-iodine diet and discontinue thyroxine medication for 3–4 weeks prior to ^131^I treatment. The ^131^I dose ranged from 3.7 to 7.4GBq (100–200 mCi) for each treatment. Posttherapy scanning (known as Rx-WBS) was performed 3 days after ^131^I treatment. Interpretation of the WBS images was conducted by two experienced nuclear medicine physicians.

### DCNN model construction

All patients underwent chest CT to examine metastatic lesions without the use of contrast media, so as not to affect subsequent radioiodine therapy. CT images were downloaded from the database in DICOM format using a picture archiving and communication system (PACS). Equipment manufactured by Philips (Amsterdam, Netherlands) and GE Healthcare (Chicago, USA) was used to generate chest CT images. All CT images were reconstructed using a medium-sharp reconstruction algorithm with a thickness of 1 mm. The objective of our research was to classify the ^131^I uptake capacity of DTC pulmonary metastatic nodules, which mainly consisted of two major steps: First, we used a pulmonary nodule detection network to extract pulmonary nodules from patient lung CT images, and then cropped the detected pulmonary nodules into fixed sizes (64 × 64). Second, the detected pulmonary nodules were fed into a deep learning classification model for further lesion differentiation.

### Pulmonary metastatic nodule detection network

For automatic and accurate pulmonary nodule detection from lung CT scans, we developed a novel method to ensure robustness in nodule detection and classification. This method is based on Faster-RCNN (18) with the feature pyramid network (FPN) (19) as the main architecture, and pulmonary nodules of size 64 × 64 were extracted. The network is divided into three modules: feature extraction (backbone + neck), region proposal network (RPN head), and proposal box re-regression and classification (region-of-interest [RoI] head). We propose a weighted patch sampling method to sample false-positive candidates and extract coarse segmentation of anatomical structures from CT. Inspired by related works on false-positive reduction (20, 21), we propose a parallel multitask RoI head for anatomical structure-aware nodule classification, which has three subheads to classify nodules or nonnodules using both nodule features and context features. The model mimics the diagnosis of a case by more than one doctor and forces the three subheads to learn from different nodule features while generating the same results. The final model benefits from both the anatomical structures and the parallel multitask RoI head. As with other methods, free-response receiver operating characteristic (FROC) analysis is employed to measure the performance of the model.

### Pulmonary metastatic nodule classification network

Image classification is a key task in the computer vision field, and CNNs have achieved state-of-the-art performance in this area. We introduce ResNeSt, a ResNet variant that incorporates a split-attention block to enhance feature representations, aiming for improved performance in image classification tasks. Specifically, the classification model consists of one stem stage and four block stages. The stem stage comprises a maximum pooling and three convolutions, with two steps for the initial convolution and pooling. The four-block stages contain the sequences three, four, six, and three blocks, respectively. The first block may include a feature dimensionality increase or down sampling. ResNeSt introduces the split-attention mechanism within its block design. The block structure divides the input into two groups, each further divided into four splits with a basic feature width of 40. Subsequently, features from the diverse groups are fused.

### Model training and testing

As is well known, complex models such as CNNs may experience overfitting, which results in suboptimal performance on data not included in the training phase. Therefore, it is necessary to properly prepare the data to evaluate the model’s performance. Based on the patient’s treatment timeline, the dataset was divided into a primary set (from January 2017 to January 2021) and an internal independent validation set (from February 2021 to June 2022). Fivefold cross-validation was applied to evaluate the classifier’s performance on the primary set (80% of the data for training and 20% for validation). To verify the model’s generalization ability, performance was further evaluated using external validation datasets 1 and 2. The performance of our deep learning model for predicting ^131^I uptake in lung metastases of DTC was compared with two other classical architectures, Inception V3 (22) and ResNet-50 (23). Receiver operating characteristic curves (ROC) were plotted, and the area under the curve (AUC), sensitivity, specificity, positive predictive value, negative predictive value, and accuracy were used to assess the model’s performance.

### Implementation and training strategy

The model is implemented by PyTorch on two Nvidia Tesla P100 GPUs. During training, each model was trained for 1,000 epochs using Adam optimization with a momentum of 0.9. The batch size was limited by GPU memory; we set it to 128 (float16) on an Nvidia GTX 1080Ti GPU. A weight decay of 0.0001 was applied. The initial learning rate was 0.0001 and was reduced to 0.00001 after half of the total number of epochs. A linear warm-up learning rate schedule was also applied during the first epoch.

### Statistical analysis

We demonstrated the diagnostic ability of the deep learning model to discriminate DTC lung metastases that are ^131^I-avid from those that are non-^131^I-avid using independent internal and external validation datasets. Accuracy, sensitivity, specificity, and positive and negative predictive values with 95% CIs were reported for our model (ResNeSt50) and two classical DCNN architectures (Inception V3 and ResNet-50). Statistical analyses were performed using the R package (version 3.6), pROC (version 1.12.1), and GraphPad Prism 7.0.

## Results

### Patient cohorts

**Figure 1:**
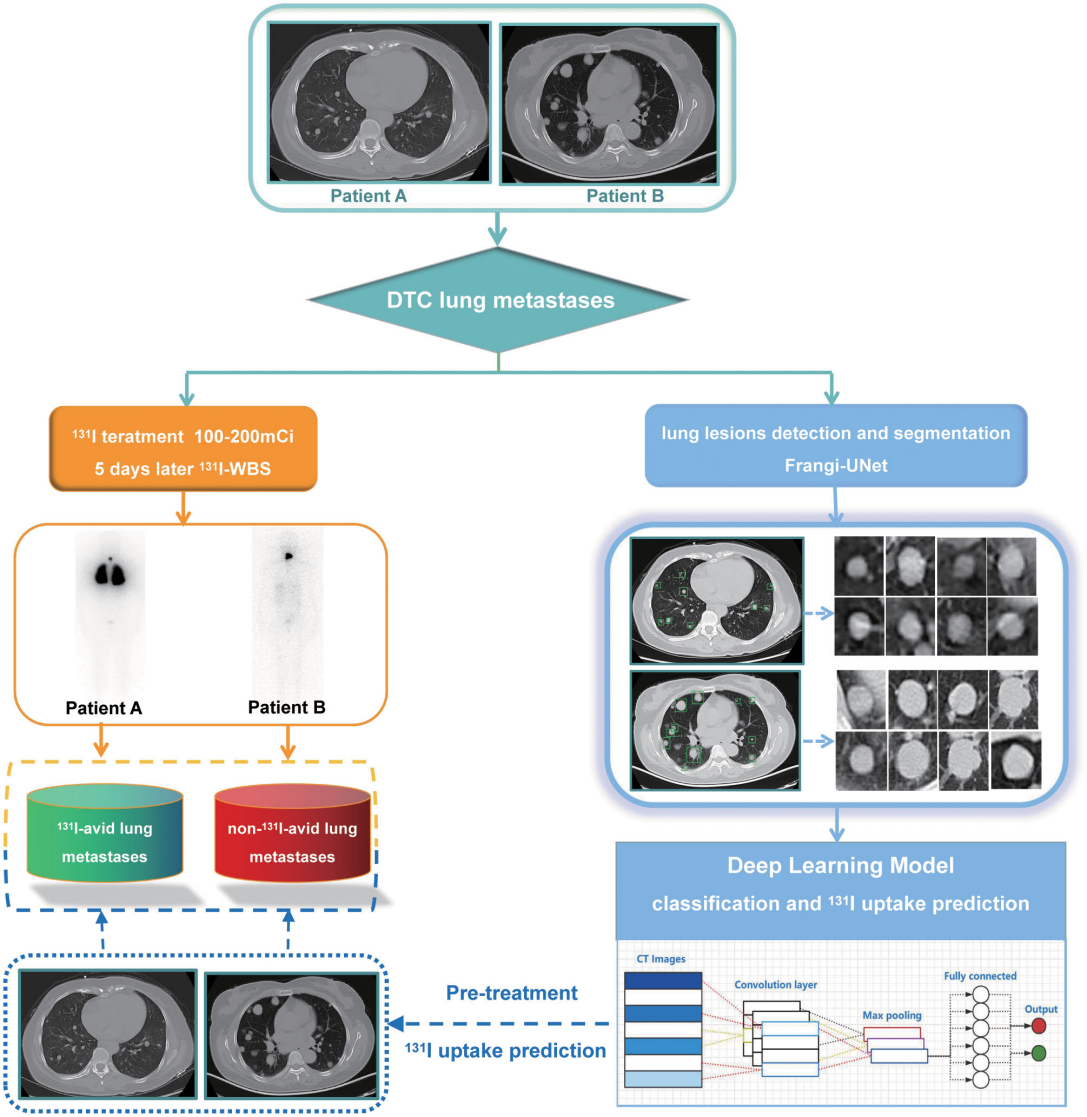
Study flowchart of procedures in the development and evaluation of deep learning models for automatically predicting ^131^I uptake in lung metastases of DTC before radioiodine therapy. CNN, convolutional neural network.

Between January 2017 and January 2021, 302 patients with DTC lung metastases were included in the primary cohort at our institution. Following a quality control evaluation, 41 patients were subsequently excluded for the following reasons: (i) patients with negative chest CT but positive ^131^I uptake on ^131^I-WBS, defined as “fine miliaric” (*n* = 11); (ii) patients with diffuse miliary pulmonary metastases (*n* = 13); and (iii) patients with low-quality chest CT images (*n* = 17). Ultimately, the primary set consisted of 20,175 images from 261 individuals: 140 patients with ^131^I-avid DTC lung metastases (10,220 images) and 121 with non-^131^I-avid (9,955 images). We conducted a fivefold cross-validation experiment on the primary set to evaluate the performance of our model. Between February 2021 and June 2022, independent validation datasets were obtained: 59 individuals for the internal validation dataset, 43 for the external validation dataset 1, and 41 for the external validation dataset 2. Baseline characteristics of the primary set and three independent validation sets are shown in **Table 1**. These datasets serve as the foundation for predicting ^131^I uptake in DTC lung metastases before radioiodine therapy. **Figure 1** illustrates the study process in a flowchart.

**Table 1:** Demographic data for 404 DTC lung metastases patients.

Characteristics	Primary set		IVD		EVD1		EVD2	
	^131^I-avid	Non-^131^I-avid	^131^I-avid	Non-^131^I-avid	^131^I-avid	Non-^131^I-avid	^131^I-avid	Non-^131^I-avid
Patients	140	121	36	23	25	18	23	18
Age^a^	42 ± 11	45 ± 12	41 ± 13	43 ± 9	38 ± 12	42 ± 10	40 ± 8	41 ± 12
Gender								
Men	56	36	9	8	8	7	7	6
Women	84	85	27	15	17	11	16	12
Pathology								
PTC	108	95	30	20	21	16	21	13
FTC	32	26	6	3	4	2	2	5
DM								
Lung	128	107	34	20	24	15	21	17
Lung + others	12	14	2	3	1	3	2	1
No. of nodes	15,507	13,891	2,897	3,051	2,632	2,281	2,755	2,360
Node size								
< 5 mm	278	169	79	91	58	43	67	41
5–10 mm	12,522	10,275	2,217	2,175	2,093	1,981	2,383	2,155
10–20 mm	2,478	3,138	537	702	433	201	254	113
> 20 mm	229	309	64	83	48	56	51	57

Unless otherwise specified, data in parentheses are percentages.

DM, distant metastatic; PD, primary dataset; IVD, internal validation dataset; EVD1, external validation dataset.

a Numbers in parentheses are the range.

### Performance of deep learning models

#### Pulmonary metastatic nodule detection

**Figure 2:**
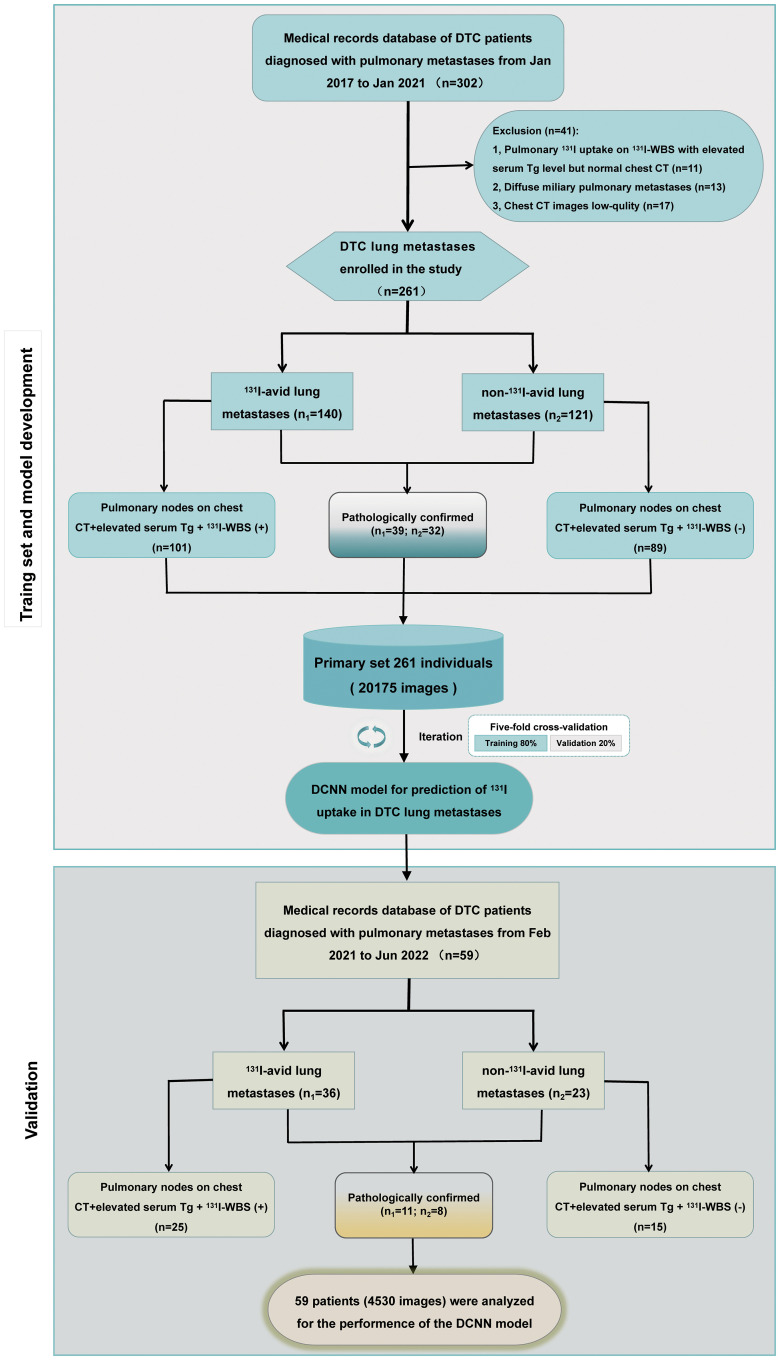
The proposed framework of a parallel multitask RoI head. A whole CT volume is fed into the preprocess module to extract coarse anatomical structures and weights. The input image patch is then sampled via the weights. The backbone and FPN extract multiscale feature maps and feed them into the RPN head. Next, the RPN head proposes RoIs to the parallel multitask RoI head. For each proposed nodule RoI (nRoI), we use a time-scaled nRoI to generate a context RoI (cRoI). The context branch focuses only on the context category classification. For each branch, three parallel subheads perform the same task. Finally, the RoI head classifies whether the proposal is a nodule based on the features of the nodule and context.

**Figure 3:**
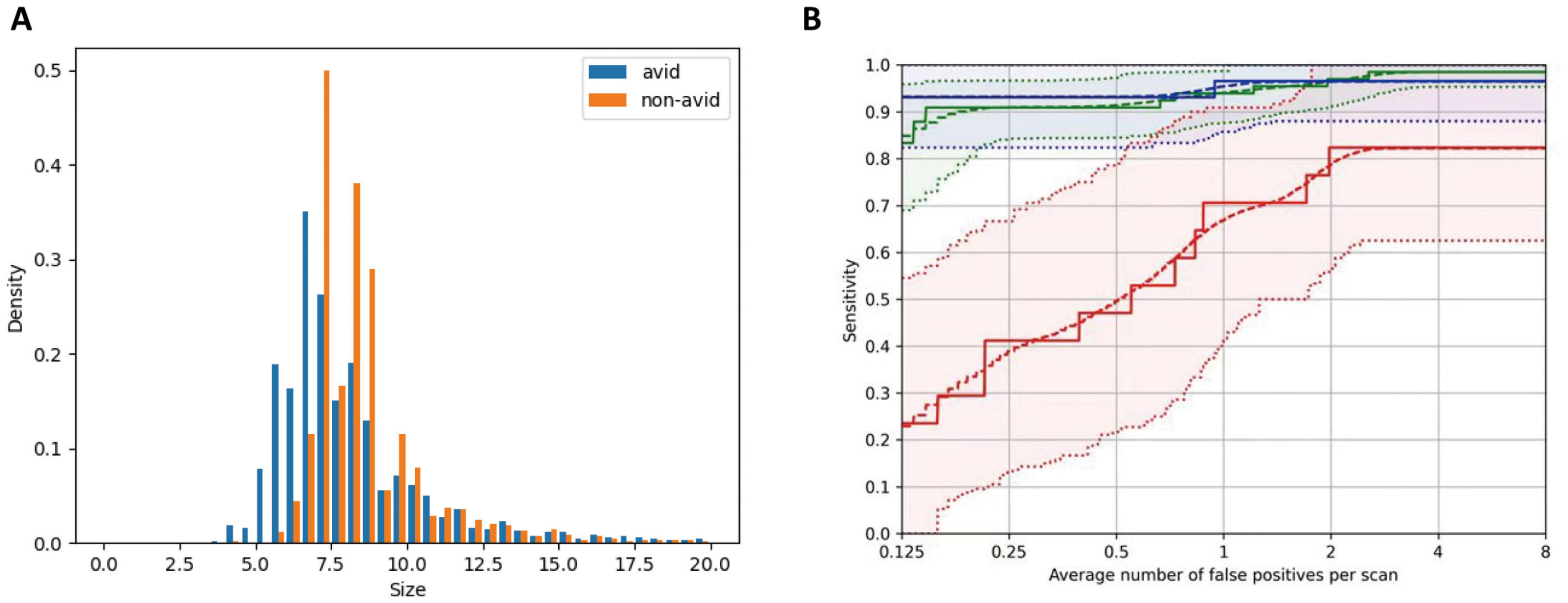
**(a)** Distribution of pulmonary nodule size. **(b)** FROC curves of different lung nodule sizes. FROC, free-response receiver operating characteristic.

In recent years, there have been significant advancements in deep convolutional neural networks for the detection of pulmonary nodules. In this study, we proposed a parallel multitask RoI head method to enhance the robustness of nodule detection. **Figure 2** shows the overall framework of our pulmonary nodule detection method. A total of 15,507 lung nodules were extracted from the ^131^I-avid group and 13,891 nodules from the non-^131^I-avid group, as summarized in **Table 1**. These pulmonary metastatic nodules were further classified based on their size: less than 5 mm (447 nodules), 5–10 mm (22,779 nodules), 10–20 mm (5,616 nodules), and larger than 20 mm (538 nodules). The distribution of pulmonary nodule sizes is shown in **Figure 3a**, with the majority of nodules falling within the 5–10 mm range.

The performance of pulmonary nodule detection was evaluated using FROC curves, which plot sensitivity against false positives (FPs) per scan (FPs/scan). FPs/scan represents the ratio of the number of false positives to the total number of cases considered for evaluation. **Figure 3b** shows the FROC curves for pulmonary nodules of different sizes: 0–5 mm, 5–10 mm, and > 10 mm, denoted by the red, green, and blue curves, respectively. The dashed lines above and below each curve indicate the upper and lower bounds of the bootstrap. Detection performance for 0–5 mm pulmonary metastasis nodules was the weakest, with a recall rate of approximately 35% at an FP/scan of 0.25. For nodules larger than 5 mm, a recall rate of 95% was achieved at an FP/scan of 0.25. This performance disparity is likely attributable to false positives arising from factors such as small blood vessels, lung walls, or rib humps.

#### Pulmonary metastatic nodule classification

**Figure 4:**
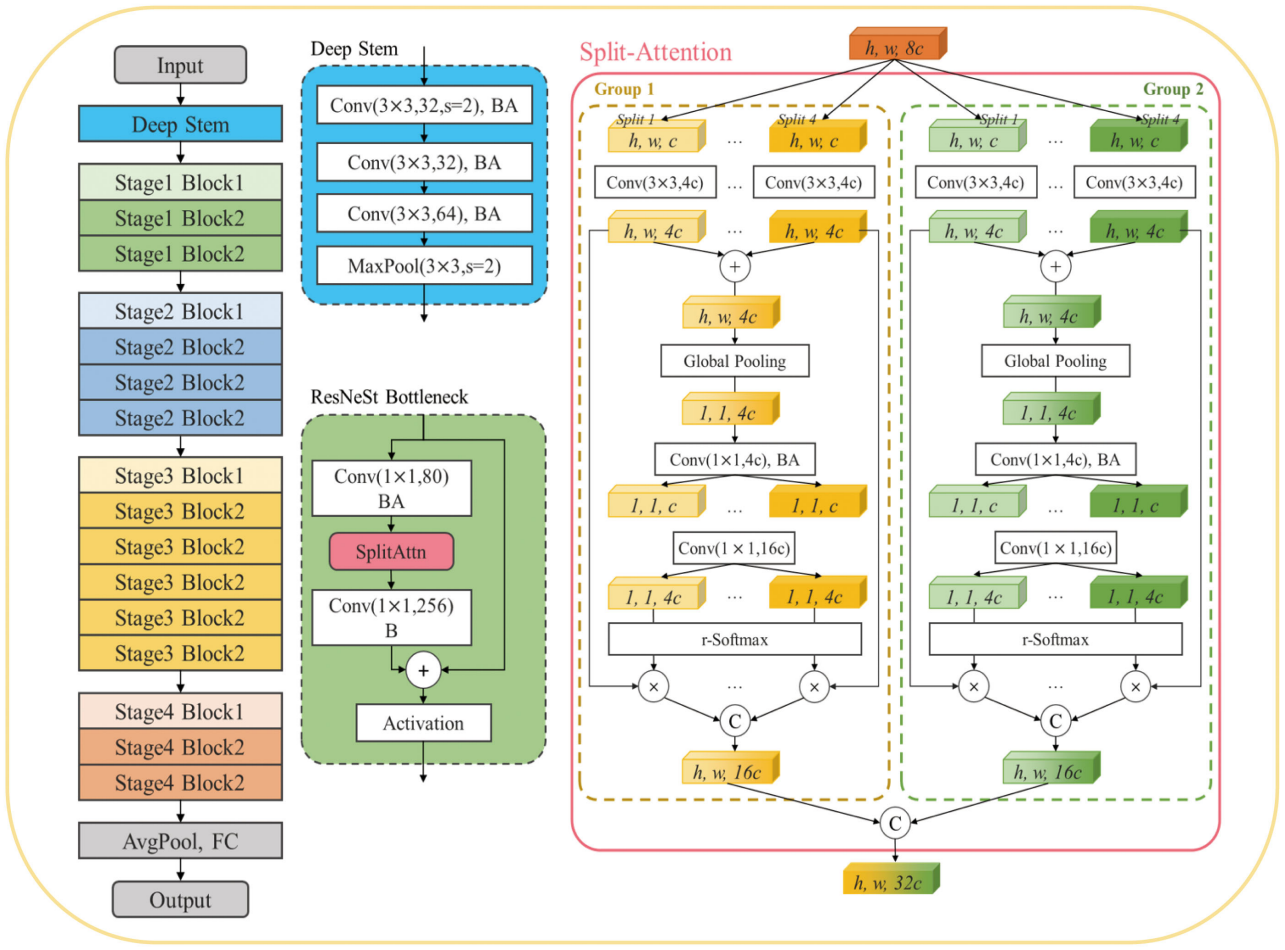
Detailed view of the split-attention unit for ResNeSt50. The deep learning classification model is based on the ResNet50 framework and includes one stem stage and four block stages. The stem stage includes three convolutions and a maximum pooling layer, with two steps for both the first convolution and pooling.

**Figure 5:**
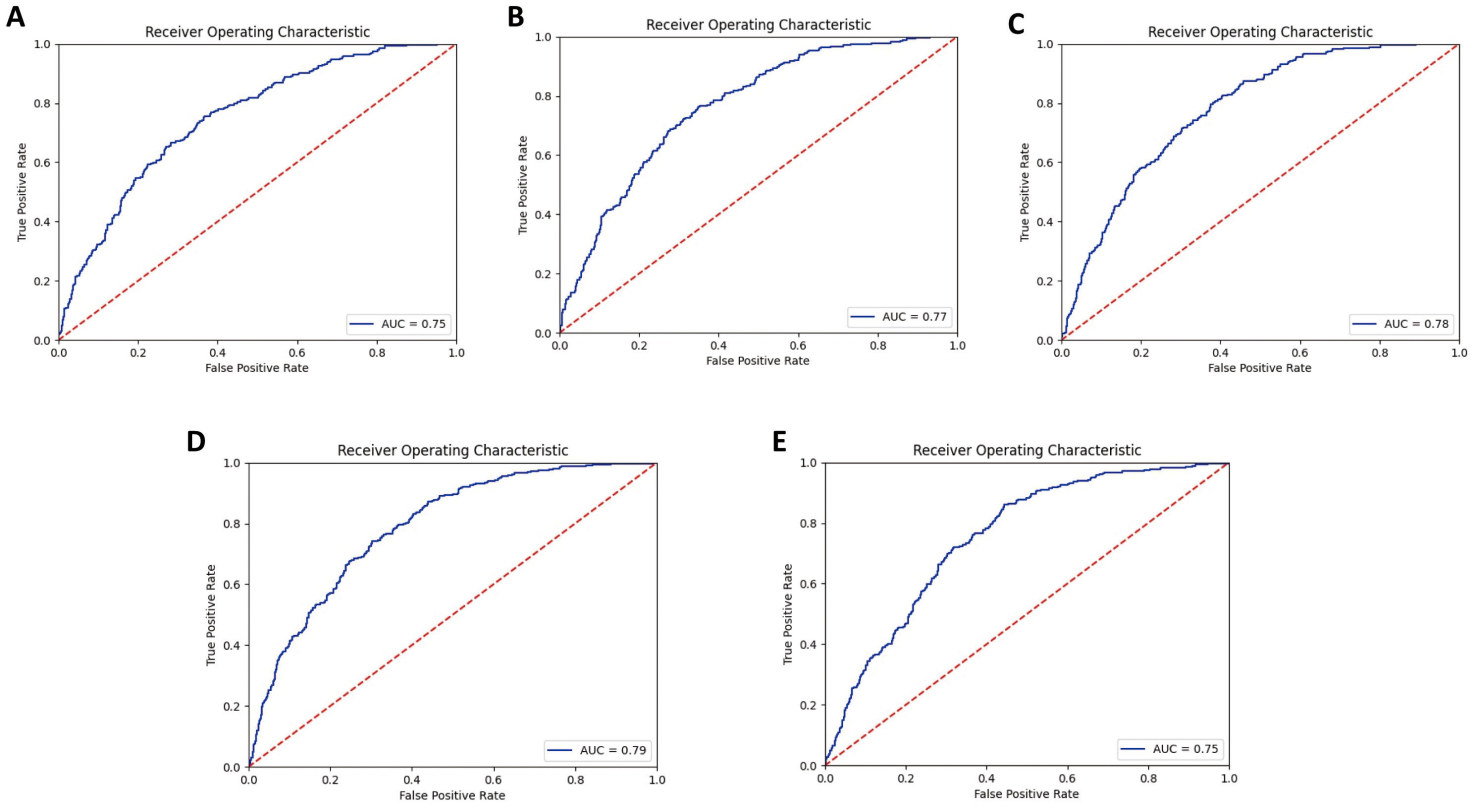
Performance of the deep convolutional neural network model to discriminate ^131^I-avid (positive ^131^I uptake) from non-^131^I-avid (negative ^131^I uptake) DTC lung metastatic modules. Receiver operating characteristic curves of the cross-validation dataset (average) **(a)**, the internal validation dataset **(b)**, the external validation dataset 1 **(c)**, and the external validation dataset 2 **(d)**.

In our study, we utilized a deep learning model called ResNeSt50 as the primary tool for discriminating between positive and negative ^131^I uptake in DTC lung metastatic nodules using chest CT images. A detailed view of the split-attention unit is shown in **Figure 4**. The model demonstrated promising predictive performance in fivefold cross-validation. During this stage, the average AUC for the ResNeSt50 model was 0.770 (95% CI = 0.767–0.775), indicating good discrimination ability. The model accuracy was 71.0% (95% CI = 70.9%–71.0%), sensitivity was 75.7% (95% CI = 70.7%–80.7%), specificity was 65.9% (95% CI = 60.2%–71.6%), positive predictive value (PPV) was 70.3% (95% CI = 65.1%–75.4%), and negative predictive value (NPV) was 71.8% (95% CI = 66.2%–77.5%). ResNeSt50 also demonstrated good performance in the independent validation datasets, with AUC values of 0.722 (95% CI = 0.716–0.725) for the internal independent validation dataset, 0.720 (95% CI = 0.691–0.749) for the Shanghai RuiJin external validation dataset 1, and 0.731 (95% CI = 0.713–0.748) for the Shanghai Tenth People’s Hospital external validation dataset 2. Compared with two other classical architectures, ResNet50 and Inception V3, ResNeSt50 achieved the best performance, as shown in **Table 2**. **Figure 5** illustrates the ROC curves for ResNeSt50 in the independent validation datasets. Overall, ResNeSt50 showed promising predictive performance in discriminating positive and negative ^131^I uptake in DTC lung metastatic nodules based on chest CT images. It outperformed other classical architectures in terms of accuracy and demonstrated good discriminative ability in both cross-validation and independent validation sets.

**Table 2:** Performance of three CNN models in the cross-validation and independent validation sets.

Findings	ResNet50	Inception V3	ResNeSt50
Cross-validation set (average)			
AUC	0.708 (0.673–0.742)	0.694 (0.662–0.718)	0.773 (0.743–0.801)
Accuracy	0.693 (0.671–0.707)	0.676 (0.653–0.698)	0.712 (0.691–0.732)
Sensitivity	0.748 (0.693–0.802)	0.731 (0.674–0.784)	0.759 (0.711–0.812)
Specificity	0.654 (0.582–0.719)	0.620 (0.543–0.688)	0.661 (0.603–0.723)
PPV	0.686 (0.634–0.739)	0.670 (0.613–0.726)	0.705 (0.652–0.758)
NPV	0.702 (0.643–0.764)	0.681 (0.623–0.746)	0.719 (0.664–0.778)
Internal validation dataset			
AUC	0.631 (0.584–0.671)	0.678 (0.634–0.721)	0.724 (0.682–0.759)
Accuracy	0.582 (0.552–0.609)	0.616 (0.592–0.641)	0.628 (0.602–0.653)
Sensitivity	0.794 (0.703–0.869)	0.814 (0.732–0.887)	0.776 (0.694–0.852)
Specificity	0.368 (0.273–0.469)	0.419 (0.324–0.513)	0.478 (0.382–0.573)
PPV	0.558 (0.472–0.638)	0.583 (0.504–0.664)	0.597 (0.512–0.683)
NPV	0.639 (0.512–0.759)	0.690 (0.573–0.808)	0.678 (0.563–0.789)
External validation dataset 1			
AUC	0.620 (0.583–0.662)	0.679 (0.637–0.723)	0.723 (0.683–0.759)
Accuracy	0.575 (0.552–0.597)	0.618 (0.593–0.642)	0.631 (0.602–0.662)
Sensitivity	0.786 (0.723–0.849)	0.820 (0.752–0.874)	0.825 (0.783–0.861)
Specificity	0.366 (0.273–0.457)	0.423 (0.342–0.498)	0.540 (0.493–0.589)
PPV	0.553 (0.476–0.639)	0.590 (0.527–0.653)	0.670 (0.613–0.719)
NPV	0.633 (0.561–0.706)	0.690 (0.643–0.733)	0.710 (0.673–0.742)
External validation dataset 2			
AUC	0.670 (0.633–0.707)	0.722 (0.682–0.749)	0.734 (0.703–0.761)
Accuracy	0.613 (0.583–0.638)	0.625 (0.603–0.641)	0.654 (0.613–0.688)
Sensitivity	0.806 (0.734–0.871)	0.769 (0.694–0.842)	0.811 (0.774–0.839)
Specificity	0.412 (0.317–0.507)	0.475 (0.382–0.561)	0.557 (0.523–0.592)
PPV	0.581 (0.507–0.661)	0.595 (0.523–0.671)	0.687 (0.646–0.724)
NPV	0.632 (0.512–0.752)	0.675 (0.563–0.788)	0.694 (0.657–0.722)

Unless otherwise specified, data are percentages, with 95% confidence intervals in brackets.

CNN, convolutional neural network; AUC, area under the curve; NPV, negative predictive value; PPV, positive predictive value.

**Figure 6:**
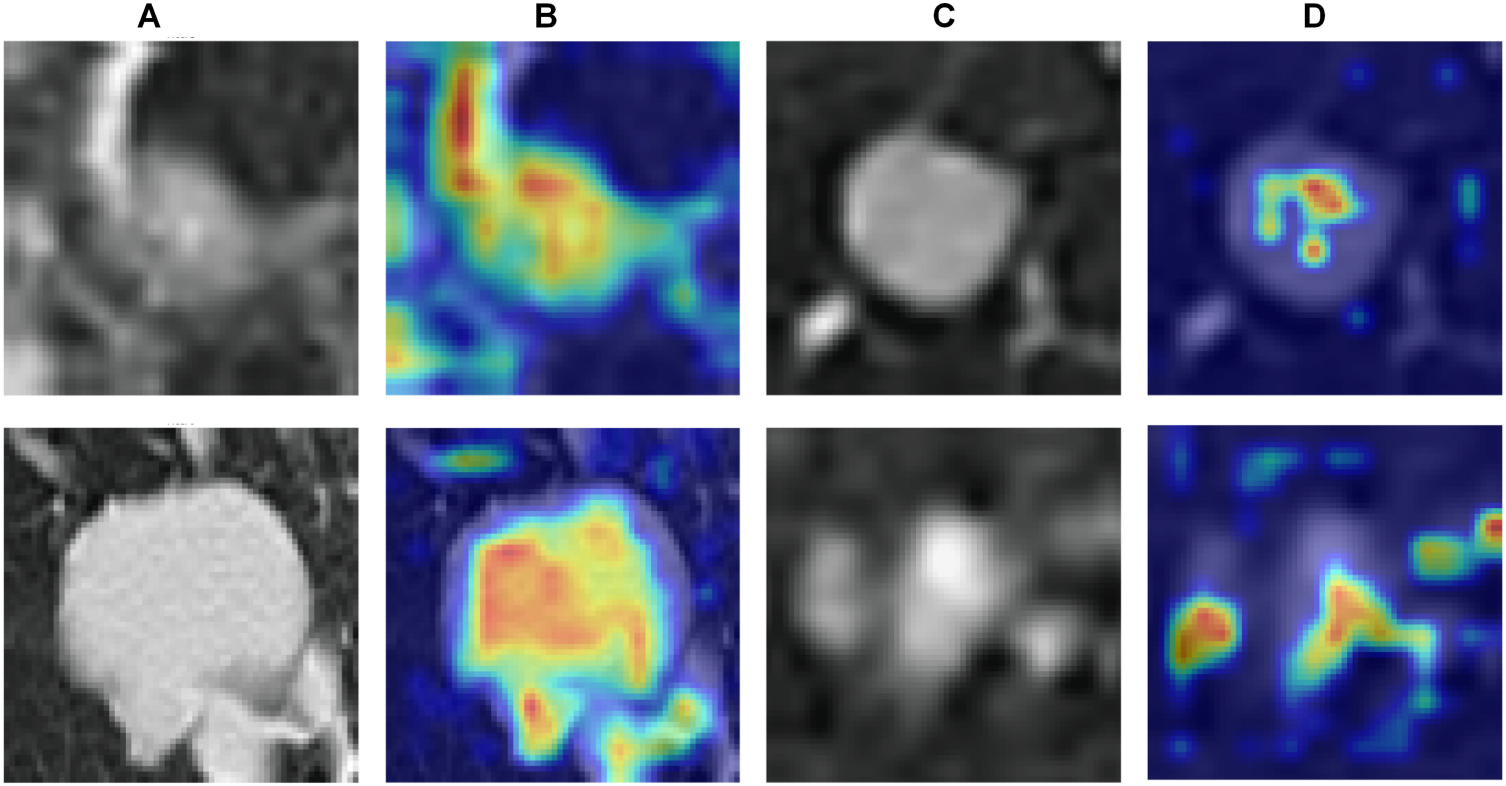
**(A**, **C)** Original images; **(B**, **D)** corresponding Grad-CAM heatmaps.

To enhance interpretability, we applied Gradient-weighted Class Activation Mapping (Grad-CAM) to visualize the regions of CT images that contributed most to the model’s predictions. As shown in **Figure 6**, the Grad-CAM heatmaps highlight that the model primarily focuses on nodule margins and heterogeneous internal areas—features consistent with radiologists’ diagnostic reasoning and known high-risk imaging characteristics. These visualizations provide intuitive evidence that the model’s decision-making process aligns with clinically meaningful regions rather than irrelevant background areas.

## Discussion

**Figure 7:**
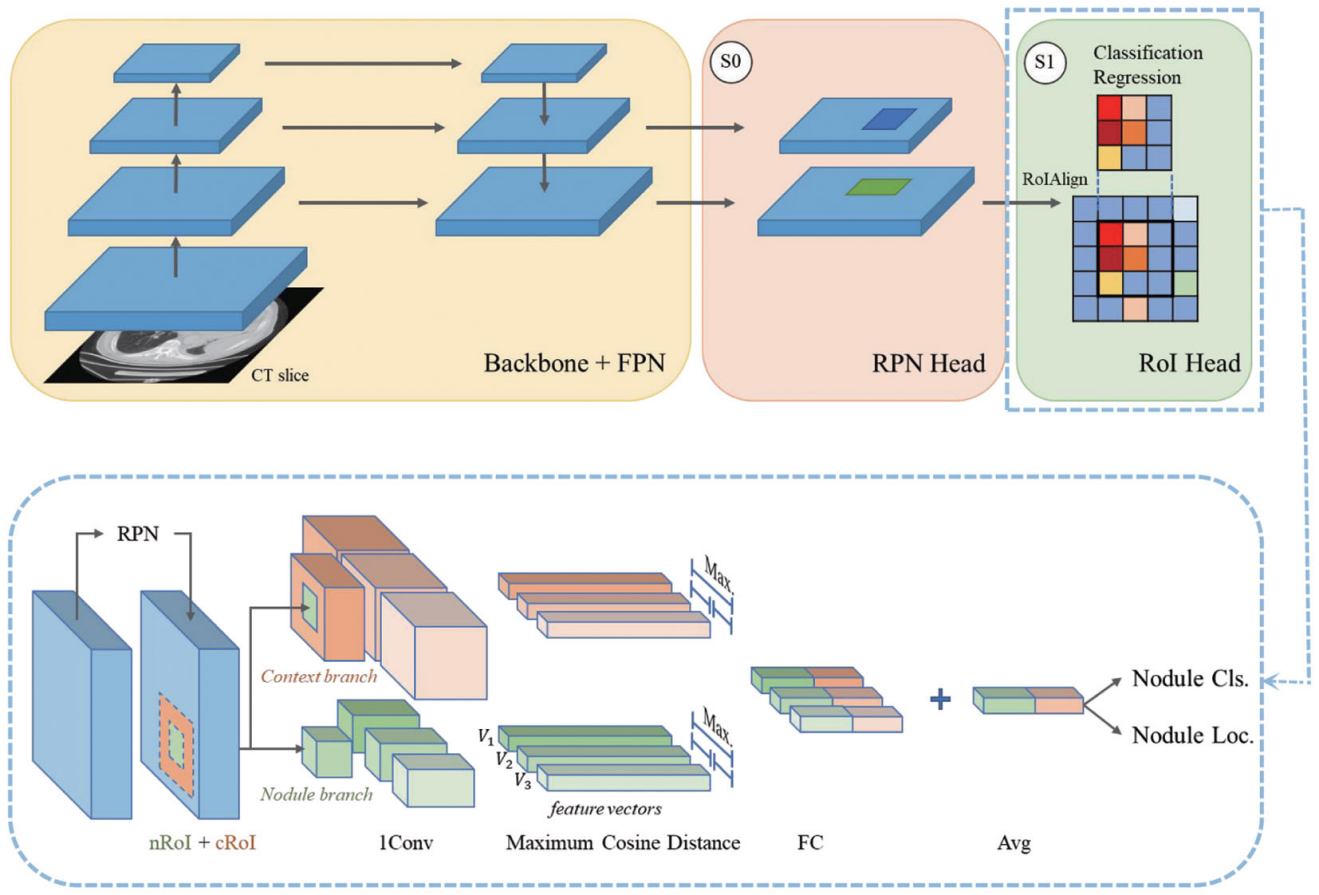
Schematic diagram of this study. The left part of the diagram shows the traditional method (posttherapeutic ^131^I whole-body scan) for diagnosing ^131^I uptake in lung metastases of DTC before radioiodine therapy, while the right half part shows the artificial intelligence method proposed in this study.

We constructed a model that automated interpreting CT images to distinguish ^131^I-avid (positive ^131^I uptake) from non-^131^I-avid (negative ^131^I uptake) DTC lung metastases prior to radioiodine therapy in a real-world setting. As a pilot study, we first created a pipeline that includes anatomical structure-aware pulmonary nodule detection via a parallel multitask RoI head to identify lung nodules from CT images. This pipeline is designed based on an actual clinical scenario and represents a novel attempt to incorporate domain expert knowledge of medical imaging into deep learning frameworks. Moreover, it achieved high sensitivity, and for lung nodules larger than 5 mm, the recall rate can achieve 95% at 0.25 FP/scan. Second, we developed a prediction model to discriminate ^131^I-avid from non-^131^I-avid DTC lung metastatic modules. The ResNeSt50 model can achieve good performance in the independent validation datasets, with AUC values of 0.722 (95% CI = 0.716–0.725) for the internal validation dataset, 0.720 (95% CI = 0.691–0.749) for external validation dataset 1, and 0.731 (95% CI = 0.713–0.748) for external validation dataset 2. These results demonstrate that this algorithm is a promising approach and may facilitate the application of deep learning techniques in the precise treatment of thyroid cancer. **Figure 7** shows a schematic diagram of our study.

The management of patients with DTC lung metastases is a clinical challenge, especially for the first radioiodine treatment, as the decision to administer high-dose ^131^I (a “blind” therapy) is problematic. We generally assess whether the patient’s lesions have iodine avidity based on the post therapeutic ^131^I-WBS, which means the patient has already received a blind high dose of ^131^I therapy. However, approximately 30% of patients with DTC pulmonary metastases lose ^131^I accumulation capacity (4), and these cases are considered RAI-refractory DTC (RAIR-DTC). In principle, patients with metastatic disease showing ^131^I accumulation should be treated with RAI therapy. In the case of RAIR-DTC, early determination is important due to the following factors: (i) unnecessary high radiation exposure; (ii) economic burden caused by ineffective ^131^I treatment; (iii) discomfort caused by discontinuation of thyroxine for 3–4 weeks (hypothyroidism) prior to RAI therapy; (iv) prolonged TSH stimulation, which may stimulate tumor growth (24); and (v) delays in other potentially more effective therapies, such as TKI administration. Therefore, identifying methods to predict ^131^I uptake in lung metastases of DTC before radioiodine therapy is crucial to selecting appropriate treatment strategies.

Based on the American Thyroid Association Guidelines, ^18^F-fluorodeoxyglucose Positron Emission Tomography/Computed Tomography (PET/CT) (^18^FDG-PET/CT), ^131^I-WBS, CT, and Tg are recommended in combination for identifying RAIR-DTC (25), but none of these tests is simple, convenient, and effective. ^18^F-FDG PET/CT is an important tool in evaluating patients with “thyroglobulin elevated but negative iodine scintigraphy Syndrome” (TENIS syndrome) and in identifying RAIR-DTC (26). An inverse relationship between ^131^I and ^18^FDG uptake (“flip-flop” phenomenon) has been described for RAIR-DTC, in which ^131^I avidity is lost while glucose metabolism increases. However, there are several drawbacks (1): ^18^F-FDG-PET/CT is valid only in patients with elevated Tg but negative ^131^I-WBS, that is, patients who have received at least one or several ineffective RAI therapies (2); metastases may develop from different tumor clones with mixed patterns of ^18^F-FDG or ^131^I accumulation (27); and (3) the cost is high and the method is unconventional. Several iodine isotopes, such as ^131^I, ^123^I, and ^124^I, play important roles in nuclear medicine, both for diagnostic purposes and therapy. Research shows that the diagnostic ^131^I (Dx) scan has a low yield in changing clinical management (28, 29). ^123^I and ^124^I are excellent diagnostic agents for whole-body scanning in patients with thyroid carcinoma and are comparable to posttreatment ^131^I scans. However, this remains a controversial topic. Lammers et al. (30) showed poor sensitivity of ^124^I PET/CT in detecting posttherapy ^131^I-positive metastases. Gauri et al. (31) and Freudenberg et al. (32) both demonstrated that a negative ^124^I PET scan has a low predictive value for a negative post-^131^I therapy scan and should not be used to exclude the option of blind ^131^I therapy. Moreover, limited availability and high cost hinder their routine application. Therefore, predicting ^131^I uptake in DTC lung metastases before radioiodine therapy is crucial but challenging. To date, AI research in this field remains largely unexplored.

In the pilot study, our work was mainly divided into two parts: detection of pulmonary metastatic nodules from lung CT scans and prediction of ^131^I uptake. To ensure robustness in lung nodule detection, we proposed an effective parallel multitask RoI head to generate nodule RoI (nRoI) and context RoI (cRoI) feature maps. This approach more closely resembles clinical diagnosis and represents a novel attempt to integrate domain expert knowledge from medical images into a deep learning method. Our study shows that the distribution of pulmonary nodule size is mainly clustered in the range of 5–10 mm, and the recall rate can achieve 95% at 0.25 FP/scan. Thus, we assert that the parallel multitask RoI head facilitates the detection of nodules from various features, and the framework demonstrates high sensitivity. Furthermore, we extracted and utilized pulmonary nodules in our prediction network and constructed a classification model based on the ResNeSt backbone (33). Specifically, we use 4s2x40d as the ResNeSt setting in this study, as it provides better training and inference speed and requires less memory. Experiments showed that ResNeSt50 achieved encouraging predictive performance in discriminating ^131^I-avid from non-^131^I-avid DTC lung metastatic modules in chest CT images. **Table 2** also shows that our proposed ResNeSt outperforms two classical architectures, Inception V3 and ResNet50, with AUC values of 0.722 (95% CI = 0.716–0.725) for the internal validation dataset, 0.720 (95% CI = 0.691–0.749) for the external validation dataset 1, and 0.731 (95% CI = 0.713–0.748) for the external validation dataset 2. The model maintained stability and generalizability across both internal and external validation datasets.

Note that, in contrast to the performance of DCNN models in other types of tumors, such as lymph node metastasis prediction from primary breast cancer (13), our model’s predictive capability is not optimal. This is mainly due to the following factors (1): As a knowledge gap, there is no available method for discriminating ^131^I-avid from non-^131^I-avid DTC lung metastatic modules. Our study is preliminary exploratory research, and the performance of the DCNN model appears comparable to that of ^18^F-FDG-PET/CT or diagnostic ^131^I scans (26–28). The principal strength of the DCNN model is its automated capability, which is accessible and freely available. (2) In our study, the data used were limited to patients’ chest CT. In addition to imaging information, many factors may affect ^131^I accumulation, such as gene mutations and age (35). However, the number of patients who have undergone gene mutation testing was limited. Future studies will include more information, such as gene mutations, which is expected to further improve the performance of the model.

The successful clinical implementation of our model may serve as a catalyst for improved patient care and resource utilization. By precisely selecting patients, it could enhance therapeutic efficiency, minimize unnecessary side effects, and allocate resources more effectively. This approach paves the way for earlier, more tailored interventions with the potential to improve long-term survival. However, translating such AI models from research to clinical practice is not without challenges. Future work must overcome significant technical and logistical hurdles, including data security, integration with institutional PACS/RIS, and the development of user-friendly interfaces. Furthermore, obtaining regulatory approval as a medical device is a critical and necessary step. Successful navigation of these challenges is a prerequisite for meaningful clinical implementation of our model. Moreover, it is important to note that this model is designed as a clinical decision-support aid, and its outputs should be interpreted within the broader clinical context by healthcare professionals. Final decisions must involve physician judgment, incorporating other relevant information and considering patient-specific factors, as upholding patient autonomy and the physician–patient relationship are key ethical considerations for AI in medicine.

Our research has several limitations. First, this is a retrospective study, and further improvement with larger, prospective studies is needed before clinical application. Second, the study included only chest CT data without considering other relevant factors, such as genetic mutations. In future work, to improve its performance, we intend to incorporate more clinical information into the artificial intelligence system. Accurate diagnoses and successful predictions can guide clinical decision-making, improve patient outcomes, and reduce the cost of cancer management. Third, the use of CT images from multiple hospitals and scanners introduced inherent variations in imaging parameters, which, despite strict quality control, may affect model performance.

In conclusion, we demonstrated that a deep learning algorithm based on CT images could potentially serve as a new tool for predicting ^131^I uptake in lung metastases of thyroid cancer before radioiodine therapy. As a pilot study, the performance of the DCNN model is encouraging. With further validation in a larger population and model calibration, our convolutional neural network-based model has strong potential to serve as an important decision-support tool in clinical applications.

## Data Availability

The original contributions presented in the study are included in the article/supplementary material. Further inquiries can be directed to the corresponding authors.
